# Impact of Computerized Order Entry to Pharmacy Interface on Order-Infusion Pump Discrepancies

**DOI:** 10.1155/2015/686598

**Published:** 2015-11-18

**Authors:** Rebecca A. Russell, David Triscari, Kathy Murkowski, Matthew C. Scanlon

**Affiliations:** Department of Pediatrics, Division of Critical Care, Medical College of Wisconsin, Milwaukee, WI 53226, USA

## Abstract

*Background*. The ability of safety technologies to decrease errors, harm, and risk to patients has yet to be demonstrated consistently.* Objective*. To compare discrepancies between medication and intravenous fluid (IVF) orders and bedside infusion pump settings within a pediatric intensive care unit (PICU) before and after implementation of an interface between computerized physician order entry (CPOE) and pharmacy systems.* Methods*. Within a 72-bed PICU, medication and IVF orders in the CPOE system and bedside infusion pump settings were collected. Rates of discrepancy were calculated and categorized by type. Results were compared to a study conducted prior to interface implementation. Expansion of PICU also occurred between study periods.* Results*. Of 455 observations, discrepancy rate decreased for IVF (*p* = 0.01) compared to previous study. Overall discrepancy rate for medications was unchanged; however, medications infusing without an order decreased (*p* < 0.01), and orders without corresponding infusion increased (*p* < 0.05).* Conclusions*. Following implementation of an interface between CPOE and pharmacy systems, fewer discrepancies between IVF orders and infusion pump settings were observed. Discrepancies for medications did not change, and some types of discrepancies increased. In addition to interface implementation, changes in healthcare delivery and workflow related to ICU expansion contributed to observed changes.

## 1. Introduction

Medication errors are known to be a significant source of risk and harm to pediatric patients [1–4], particularly in neonatal and pediatric intensive care units (PICUs) [5–8]. A study looking at prescribing errors intercepted by pharmacists in a maternity and children's hospital found the risk of prescribing errors to be fourfold higher in pediatric patients than in the maternity population (1,391 errors in 61,458 orders versus 644 errors per 119,333 orders) [9]. They found that intercepted errors in pediatric patients were most commonly related to dosing, with detection of doses 1.5- to 10-fold higher than recommended. Finally, the use of intravenous (IV) medications has also been identified as a specific source of medical risk [10, 11].

In response to the errors and harm associated with medication infusions, the adoption of a range of healthcare information technologies including computer order physician entry (CPOE), bar coded medication administration, and “smart” infusion pumps has been recommended [12–14]. While these technologies have demonstrated the capacity to decrease the frequency of errors [15–17], results are mixed in studies trying to document reduction in harm. While some have been able to show a decrease in mortality [18, 19], others have failed to demonstrate efficacy of the technology or reduction in harm [20–26]. Notably, others have uncovered associations between CPOE and new and unanticipated errors as well as harm [27–30].

The same authors as the present paper conducted a study in 2007 measuring discrepancies between medication orders for infusions entered into a CPOE system and the medication (or intravenous fluid (IVF)) actually being infused at the bedside within a PICU [31]. Rates of discrepancy were calculated and discrepancies were categorized by type. A high frequency of discrepancies between CPOE orders for medications and programmed settings on “smart” infusion pumps at the bedside (24%) and between intravenous fluids and programmed pump settings (42%) was observed. We concluded that the discrepancies were most likely related to interactions between providers performing the tasks of ordering intravenous medications and fluids and the programming of the infusion pumps and that more intensive understanding of these interactions was essential to the process of risk mitigation and elimination.

Since the time of the original study, a bidirectional interface between the CPOE and pharmacy systems was established at the study site. This allowed orders entered by providers to go directly to the pharmacy system for verification rather than being printed and reentered into the pharmacy system by a pharmacist prior to order verification. The bidirectional interface is an example of a “closed-loop” system. Closed-loop medication management systems automate steps in the complex process of medication delivery in an effort to reduce the number of opportunities for mistakes. Relatively little has been published about the performance of these systems. One study in 2012 demonstrated a decrease in time from order placement to administration of antibiotics following implementation of a closed-loop order-processing system [32]. Another study examined the effects of closed-loop electronic prescribing, automated dispensing, barcode patient identification, and electronic medication administration record and found a decrease in prescribing and administration errors [33]. We were interested in examining the impact of a bidirectional interface between CPOE and pharmacy systems within a PICU on the frequency and types of discrepancies between orders for medication and IVF infusions and settings on bedside infusion pumps. We hypothesize that the frequency of discrepancies will be less but that the observed changes in rates and types of discrepancies will not be attributable to the closed-loop system alone. The PICU also underwent expansion and relocation in the interim, and we suspect that changes in workflow may be in part responsible for any observed decreases in discrepancies.

## 2. Materials and Methods

### 2.1. Study Design

This was an uncontrolled before and after study utilizing a prospective, observational design. The study was performed in a 72-bed PICU with approximately 1800 admissions per year within a freestanding children's hospital located in the Midwest. Patients admitted to this PICU include children ranging from one day to over 18 years of age, with a wide range of disease processes including trauma, cardiothoracic surgery, respiratory failure, metabolic disease, and sepsis. Trained PICU nurses perform infusion pump programming. Orders for IV medication infusions and IVF may be entered into the CPOE system by attending physicians, trainees (fellows and resident physicians), and nurse practitioners and physician assistants. Data were gathered over a period of seventeen weekdays. Between study periods, the PICU also underwent relocation and expansion from 30 beds to 72 beds. Approval from the hospital Institutional Review Board was obtained prior to any data collection.

### 2.2. Technologies

The CPOE system, Sunrise Clinical Manager Version 4.5 by Eclipsys Technologies Corporation, was implemented in September of 2000. The smart infusion pumps (Smith-Medex) were implemented in 2006. A closed-loop system consisting of a bidirectional interface between CPOE and pharmacy systems was established in September of 2008.

### 2.3. Data Collection

Intravenous medication infusions were defined as medications requiring the use of an infusion pump. Only medications given as continuous IV infusion were included. Medications given via infusion pump were excluded if dosing was intermittent, a change from methodology of the previous study. Intravenous fluids were defined as total parenteral nutrition (TPN), lipids, and crystalloid infusions given continuously via infusion pump.

Data were simultaneously collected from the medication orders in the CPOE system and the bedside infusion pumps by trained observers once a day over a period of seventeen days in August of 2010. One observer recorded date, time, bed number, each infused medication or fluid, and corresponding dose or rate. A second observer simultaneously recorded existing orders by bed space, capturing the date and time of observation, and each ordered medication or fluid with its respective dose or rate. No patient-related information was collected during this study.

### 2.4. Analysis

A line-by-line comparison of order observation data with the pump observation data was performed, matched by time and bed number. Observations occurring more than fifteen minutes apart were excluded to minimize the effect of any interim changes to order or pump. For each observed medication infusion or IVF, analysis began with verifying the presence of a corresponding order. For those that had a corresponding order, the medication doses or IVF rates that were ordered were compared to those observed to be infusing at the bedside. The frequency of discrepancies was established and then categorized by type.

Discrepancies were defined as follows: a medication or fluid found to be infusing without a corresponding order was categorized as an* unauthorized medication* or* unauthorized fluid*. When no medication or fluid infusion was observed despite the presence of an active order, this discrepancy was categorized as an* omitted medication* or* omitted fluid*. Finally, when the medication or fluid infusing at the bedside was observed to differ in dose (for medications) or rate (for fluids) when compared to the active order in CPOE, this discrepancy was categorized as a* wrong medication dose* or* wrong fluid rate* [34]. Any difference in dose (for medications) or rate (for IVF) between the computerized order and the bedside pump setting was counted as a discrepancy regardless of the magnitude of the difference.

Proportions of discrepant medications and fluids for the two study periods (original study in 2007, current study in 2010) were compared. *p* values were calculated using two-proportion *z* test, and *p* values < 0.05 were considered significant.

## 3. Results

A total of 303 observations of medication infusions and 152 observations of IVF were completed during the study period. This constituted 79 fewer IVF observations compared to the study in 2007. The number of medication observations was similar (296 observations in 2007 versus 303 observations in the current study). No observations required exclusion based on the predetermined 15-minute time window for orders and programming. During this study period 54 of 303 (18%) observations of medication infusions revealed order-programming discrepancies, while 46 of the 152 (30%) observations of IVF revealed order-infusion pump discrepancies (Tables 1 and 2). The decrease seen in medication infusion discrepancies did not reach statistical significance (*p* = 0.05). However, this represented an overall decrease in the discrepancy rate for the IVF group, which included TPN, lipids, and crystalloid infusions, following implementation of the bidirectional interface (*p* = 0.01).

The decrease in discrepancies between orders and IVF was due in large part to a significant decrease in discrepancies for TPN. Ten of 55 (18%) TPN observations had discrepancies, down from a rate of 50% in the prior study (*p* < 0.01) (Table 2). There were no significant changes in overall rates of discrepancy for crystalloid or intralipid infusions. However, the proportion of unauthorized fluid discrepancies (IVF infusing without a corresponding order) decreased significantly (32% to 15%, *p* < 0.05) (Table 3).

Although the overall discrepancy rate for medications did not change following implementation of the pharmacy-order entry interface, the types of discrepancies that were observed did change. For example, unauthorized medications (medications infusing without a corresponding order) accounted for 60% of the medication discrepancies in the previous study and decreased to 4% (*p* < 0.01) in the current study (Table 3). On the other hand, we observed significant increases in the proportion of omitted medications (order present but no infusion) and medications infusing at the wrong dose (*p* < 0.05 for both).

Similar to the previous study, when discrepancies in medication infusions were examined by type of medication, the greatest frequency of discrepancies occurred within cardiovascular medication group (27%, *n* = 35 of 128 observations) (Table 1). Discrepancies between orders and pump programing for milrinone and epinephrine infusions increased between study periods (*p* < 0.01 for both). Finally, the anticoagulant group, comprised entirely of heparin infusions for the current study, saw a reduction in discrepancy rate from 46% to 9% (*p* < 0.01).

## 4. Discussion

In a study performed in 2007, we observed a high frequency of discrepancies between medication and intravenous fluid orders within a CPOE system and programmed settings on infusion pumps at the beside. Subsequently, a bidirectional interface between pharmacy and order entry systems was established, and the ICU underwent expansion and relocation. Our current study examines the changes in the number and types of discrepancies detected. Contrary to our hypothesis, while the rate of discrepancies decreased for IVF, the overall frequency of discrepancies among medication infusions did not decrease to a level of statistical significance between study periods. While sample size certainly could alter this finding, given the strong push for adoption of healthcare information technologies, we believe these results are still relevant. Furthermore, our analysis suggests that the observed decreases in discrepancies are not solely attributable to the technology and that workflow and other factors are partly responsible for the observed changes. For example, fewer unauthorized medication discrepancies were observed. We suspect that this change was related to the impact of the bidirectional interface on the workflow for verbal requests for new medications: previously, if a provider requested that a new medication be made urgently, the pharmacist could deliver the medication to the bedside but was unable to reconcile the order. Unless the provider immediately entered the order in CPOE, a discrepancy characterized as an unauthorized medication was created. The bidirectional interface allowed the pharmacist to immediately reconcile the verbal order (an order communicated verbally from a physician to a pharmacist). Similarly, the decrease in discrepancies for anticoagulants could be explained by the same mechanism as that just described wherein pharmacists utilize the bidirectional interface to reconcile verbal orders.

While unauthorized medications decreased, there was an increase in omitted medications as well as wrong dose discrepancies. We suspect that a change in workflow is the reason for the increase in observed omitted medication discrepancies. Around the time of PICU relocation, anesthesiologists began placing orders for cardiovascular and sedation/analgesia medications they anticipated would be used intraoperatively, such as an epinephrine or fentanyl infusion, the day prior to scheduled surgery. As such, patients in PICU awaiting surgery would have orders for these infusions “halted at bedside” in the CPOE system but no infusion present until they returned from the operating room the following day. We believe this was responsible for many of the omitted medication discrepancies.

The rate of discrepancies between orders and pump settings decreased for fluids, due in large part to a decrease in discrepancies within the total parenteral nutrition subgroup. We have not identified a mechanism by which implementation of the bidirectional interface could account for this decrease. Rather, a change in workflow on rounds is more likely responsible for this decrease. Between study periods the PICU underwent expansion and relocation. In this new environment pharmacy and dietary presence on rounds increased, resulting in greater collaboration among pharmacists, dieticians, and the providers responsible for ordering TPN. Previously, a single patient's TPN may have been reordered more than once on a given day due to modifications in the composition ordered. We suspect that the described change in workflow allowed for consensus on the desired TPN composition, resulting in fewer total TPN orders and therefore fewer opportunities for discrepancy.

Little is known about the effects of implementation of systems like this bidirectional interface or other types of closed-loop systems. Our study enhances and expands on the results of two previously published studies. One found a decrease in mean time from order to administration of IV antibiotics after closed-loop implementation in patient care areas such as general medicine and cardiology [32]. However, due to a small number of observations in intermediate and intensive care areas, the study was underpowered to detect a significant difference in these areas that were examined in our study. The second study found a decrease in medication administration errors (MAEs) for non-IV medications following closed-loop implementation but increased pharmacy and medical staff time to perform tasks [33]. However, medications given via IV infusion, the subject of our investigation, were excluded from this study. While these studies support the use of closed-loop systems, like ours they were not designed to show an improvement in patient outcome or a decrease in patient harm.

This study has several limitations. First, as mentioned, the study was not designed to detect harm, and endpoints such as frequency of adverse events or length of hospital stay were not captured. Whether the changes in discrepancies were associated with changes in the incidence of harm to patients or with improvement in other measures of quality of care cannot be determined. Also, as an uncontrolled before and after study, the ability to draw conclusions about the causes of observed changes is limited and part of the analysis is purely subjective. Finally, while the sample size allowed for detection of differences in discrepancy rates within certain groups of medications and IVF, it is possible that the other differences could have been detected with greater numbers in some of the subgroups.

## 5. Conclusions

Fewer discrepancies between IVF orders and programmed settings on bedside infusion pumps were observed following implementation of a bidirectional interface between pharmacy and order entry systems in a PICU setting. However, discrepancies between medication orders and pump settings did not decrease. Furthermore, subjective analysis of causation suggests that changes in workflow and other factors are likely to have contributed as much to the observed changes as did the bidirectional interface. Study design precluded investigation of any potential association between the changes in discrepancies and changes in patient outcomes or harm. Demonstrating such an association is essential in the assessment of the value and efficacy of safety technologies.

## Figures and Tables

**Table 1:** Discrepancies by medication category and subtype.

Medication type	Discrepancies/total observations for each type of medication		*p* value
Study year	2007	2010	
All	72/296	54/303	NS
Cardiovascular	10/34	35/128	NS
Milrinone	1/48	10/52	<0.01
Epinephrine	5/29	18/36	<0.01
Amiodarone	0	1/12	NS
Norepinephrine	1/9	5/11	NS
Prostaglandin	0	1/10	NS
Epoprostenol	0	0/6	NS
Nitroprusside	0/1	0/1	NS
Analgesia/sedation	6/58	6/71	NS
Fentanyl	4/20	3/30	NS
Dexmedetomidine	0/7	1/13	NS
Ketamine	0/8	0/11	NS
Midazolam	1/9	1/9	NS
Sufentanil	1/11	0/6	NS
Pentobarbital	0	1/2	NS
Concentrated electrolytes	7/15	1/11	NS
25% albumin	0	0	NS
3% normal saline	0	0/4	NS
Calcium gluconate	4/12	1/2	NS
Magnesium sulfate	2/2	0	NS
Potassium chloride	1/1	0	NS
Anticoagulants	30/65	4/44	<0.01
Heparin	30/65	4/41	<0.01
Antithrombin III	0	0/3	NS
Miscellaneous	5/44	8/49	NS
Furosemide	1/34	0/33	NS
Vecuronium	1/7	4/8	NS
Insulin	0	3/3	NS
Aminophylline	0	0/2	NS
Cisatracurium	0	0/2	NS
Terbutaline	0	1/1	NS

*p* value: proportion of medication type discrepant in 2007 versus 2010 using *z* test of two proportions.

**Table 2:** Discrepancies by fluid type and subcategory.

Fluid type	Discrepancies/total observations by fluid type		*p* value
Study year	2007	2010	
All	97/231	46/152	0.01
Crystalloid	60/123	17/42	NS
D5W	35/83	13/24	NS
D10W	15/19	2/3	NS
Normal saline	1/2	2/4	NS
Lactated ringers	1/1	0	NS
Nutrition	37/108	29/110	NS
TPN	22/44	10/55	<0.01
Intralipid	15/64	19/50	NS

*p* value: proportion of fluid type discrepant in 2007 versus 2010 using *z* test of two proportions.

**Table 3:** Types of discrepancy.

	Omitted medications *n*/total discrepancies of each medication type		*p* value	Unauthorized medications *n*/total discrepancies of each medication type		*p* value	Wrong dose *n*/total discrepancies of each medication type		*p* value
Study year	2007	2010		2007	2010		2007	2010	
All medications	19/72	35/54	<0.05	43/72	2/54	<0.01	10/72	17/54	<0.05
Cardiovascular	3/10	22/35	NS	3/10	2/35	<0.05	4/10	11/35	NS
Analgesia/sedation	0/6	3/6	<0.05	1/6	0/6	NS	5/6	3/6	NS
Anticoagulants	16/30	1/4	NS	14/30	0/4	NS	0/30	3/4	<0.05
Electrolytes	0/7	1/1	<0.05	7/7	0/1	0.03	0/7	0/1	NS
Miscellaneous	0/5	8/8	<0.05	4/5	0/8	0.02	1/5	0/8	NS

	Omitted fluid *n*/total discrepancies of each fluid type		*p* value	Unauthorized fluid *n*/total discrepancies of each fluid type		*p* value	Wrong fluid rate *n*/total discrepancies of each fluid type		*p* value

Study year	2007	2010		2007	2010		2007	2010	
All fluids	24/97	16/46	NS	31/97	7/46	<0.05	42/97	23/46	NS
Crystalloid	8/60	1/17	NS	26/60	4/17	NS	26/60	12/17	<0.05
Hyperalimentation	16/37	15/29	NS	5/37	3/29	NS	16/37	11/29	NS

*p* value: proportions of discrepancies in 2007 versus 2010 using *z* test of two proportions.
